# Risk factors for perioperative ischemic stroke in cardiac surgery

**DOI:** 10.5935/1678-9741.20150032

**Published:** 2015

**Authors:** Mário Augusto Cray da Costa, Maria Fernanda Gauer, Ricardo Zaneti Gomes, Marcelo Derbli Schafranski

**Affiliations:** 1 Departamento de Medicina da Universidade Estadual de Ponta Grossa (UEPG), Ponta Grossa, PR, Brazil and cardiovascular surgeon of Santa Casa de Misericórdia de Ponta Grossa, Ponta Grossa, PR, Brazil.; 2 Universidade Estadual de Ponta Grossa (UEPG), Ponta Grossa, PR, Brazil.

**Keywords:** Stroke, Cardiac Surgical Procedures, Carotid Artery Diseases

## Abstract

**Objective:**

The purpose of this study was to evaluate the risk factors for ischemic stroke in patients undergoing cardiac surgery.

**Methods:**

From January 2010 to December 2012, 519 consecutive patients undergoing cardiac surgery were analyzed prospectively. The sample was divided into two groups: patients with stroke per and postoperative were allocated in Group GS (n=22) and the other patients in the group CCONTROL (n=497). The following variables were compared between the groups: gender, age, carotid stenosis ≥ 70%, diabetes on insulin, chronic obstructive pulmonary disease, peripheral arteriopathy, unstable angina, kidney function, left ventricular function, acute myocardial infarction, pulmonary arterial hypertension, use of cardiopulmonary bypass. Ischemic stroke was defined as symptoms lasting over 24 hours associated with changes in brain computed tomography scan. The variables were compared using Fisher’s exact test, Chi square, Student’s t-test and logistic regression.

**Results:**

Stroke occurred in 4.2% of patients and the risk factors statistically significant were: carotid stenosis of 70% or more (*P*=0.03; OR 5.07; IC 95%: 1.35 to 19.02), diabetes on insulin (*P*=0.04; OR 2.61; IC 95%: 1.10 to 6.21) and peripheral arteriopathy (*P*=0.03; OR 2.61; 95% CI: 1.08 to 6.28).

**Conclusion:**

Risk factors for ischemic stroke were carotid stenosis of 70% or more, diabetes on insulin and peripheral arteriopathy.

## Abstract (pt)

**Objetivo:**

O objetivo do presente trabalho foi avaliar os fatores de risco para acidente vascular encefálico isquêmico em pacientes submetidos à cirurgia cardíaca.

**Métodos:**

Entre janeiro de 2010 e dezembro de 2012, foram analisados prospectivamente 519 pacientes consecutivos submetidos à cirurgia cardíaca. A amostra foi dividida em dois grupos: os pacientes com acidente vascular encefálico isquêmico (AVEi) trans e pós-operatório foram alocados no grupo GAVEi (n=22) e os demais pacientes no grupo CControle (n=497). As seguintes variáveis foram comparadas entre os grupos: sexo, idade, estenose carotídea ≥70%, diabetes em uso de insulina, doença pulmonar obstrutiva crônica, arteriopatia periférica, função renal, angina instável, função do ventrículo esquerdo, infarto agudo do miocárdio recente, hipertensão arterial pulmonar, uso de circulação extracorpórea. Acidente vascular encefálico isquêmico foi definido como presença de sintomas de duração maior que 24 horas associados à alteração em tomografia de crânio. As variáveis foram comparadas, por meio do teste exato de Fisher, Qui quadrado, teste t de Student e regressão logística.

**Resultados:**

Verificou-se a ocorrência de acidente vascular encefálico isquêmico em 4,2% dos pacientes e os fatores de risco estatisticamente significativos foram: estenose carotídea de 70% ou mais (*P*=0,03; OR 5,07; IC 95%: 1,35 a 19,02), diabetes em uso de insulina (*P*=0,04; OR 2,61; IC 95%: 1,10 a 6,21) e arteriopatia periférica (*P*=0,03; OR 2,61; IC 95%: 1,08 a 6,28).

**Conclusão:**

Foram fatores de risco para acidente vascular encefálico isquêmico: estenose carotídea de 70% ou mais, presença de diabetes em uso de insulina e presença de arteriopatia periférica.

**Table:** 

**Abbreviations, acronyms & symbols**	
AMI	Acute myocardial infarction
CABG	Coronary artey bypass surgery
CC	Creatinine clearance
COPD	Chronic obstructive pulmonary disease
CPB	Cardiopulmonary bypass
CS	Carotid stenosis
CT	Computed tomography
CVA	Cerebrovascular accident
ECST	European Carotid Surgery Trial
EF	Ejection fraction
EuroSCORE	European System for Cardiac Operative Risk Eva-luation
IS	Ischemic stroke
MAP	Mean arterial pressure
NYHA	New York Heart Association
PAH	Pulmonary arterial hypertension

## INTRODUCTION

The cerebrovascular accident (CVA) is a major cause of mortality, permanent neurological damage and increased health expenditures^[1,2]^. American Heart Association data cited by Santos et al.^[3]^ show that in the United States, 18% of the causes of death from cardiovascular cause are due to occurrence of CVA. In the context of heart surgery, ischemic stroke (IS) is one of the largest and most feared perioperative complications, with an incidence is 2% for all cardiac surgeries according Naylor & Brown^[1]^. When it comes to coronary artey bypass surgery (CABG) the incidence may reach up to 6%, and the risk grows to 12% in patients with severe carotid stenosis (CS)^[2-7]^.

The timing of occurrence of perioperative stroke has a bimodal distribution, approximately 45% of the events are identified on the first day after surgery, in this group are the intraoperative events and those occurring in the first hours after surgery and 55% occur after recovery from anesthesia, the second day onward, thus being postoperative events. Early embolism is due to manipulation of the heart and aorta or particles transported by cardiopulmonary bypass (CPB), the later events may be related to atrial fibrillation, myocardial infarction, low output and hypercoagulability. Patients with carotid stenosis (CS) has increased incidence of ischemic stroke, but contrary to expectations, even in patients with CS, the leading cause of ischemic stroke is the embolism, accounting for 62% of ischemic events^[2,8,9]^.

Are risk factors for ischemic stroke age greater than 70 years, female gender, hypertension, diabetes mellitus, renal failure, smoking, chronic obstructive pulmonary disease (COPD), peripheral artery disease, ejection fraction of 40%, ischemic stroke history or previous transient ischemic attack, carotid stenosis, aortic calcification, surgery with CPB, prolonged cardiopulmonary bypass time, emergency surgery and others^[3-5,8,10,11]^.

Various strategies have been used to reduce the occurrence of perioperative stroke like avoiding low debit, early treatment of arrhythmias, prevent manipulation and aortic cannulation, use of membrane oxygenators and arterial filters, avoid hyperglycemia, maintain good brain oxygenation, off-pump surgery and prophylactic treatment of carotid lesions using stents or carotid endarterectomy in patients with severe stenosis of the carotid^[2]^. But it is still debated in the literature if the use of stents or carotid endarterectomy in asymptomatic patients actually prevents the perioperative ischemic stroke, because it is believed that most of the ischemic complications is related to embolism of aortic plaques and that the presence of severe carotid atherosclerosis is another ischemic events marker than its etiology, because there is no perfect correlation between ischemic stroke and the plaque side in many cases and more than half of cases of perioperative ischemic stroke has no evidence of carotid disease^[2]^.

Naylor et al.^[12]^ published in 2002 that 50% of cases of IS did not occur during occurrence of severe carotid stenosis, and 60% of cerebral infarctions could not be attributed to carotid disease. Still, in 2011, Naylor & Brown^[1]^ published systematic review and meta-analysis showing that the incidence of ipsilateral stroke in asymptomatic patients with unilateral CS between 50 and 99% was 2% and any stroke was 2.9%. In more severe lesions with 70-99% obstruction or 80-99% there was no increased incidence of ischemic stroke. In bilateral lesions the risk increased to 6.5%, but when endarterectomy was performed concomitant with revascularization the incidence of stroke in the opposite side was still 5.7%. The authors argue that the carotid lesions may be more marker than cause of stroke.

The interest in the use of off-pump surgery in order to reduce stroke has growing, Sá et al.^[13]^, through a meta-analysis with 13,524 patients, found that off-pump surgery has reduced the incidence of postoperative stroke in 20.7%, similarly to meta-analysis findings with 3,996 patients, developed by Sedrakyan et al.^[14]^. But since often off-pump surgery can not be ignored, evolution of cannulas and filter devices in the ascending aorta has been used with some advantages. Strategies to prevent aortic clamping with venous grafts anastomosed proximally to the mammary arteries are used in hostile aortas in order to minimize the risk of embolization of aortic plaques^[8]^.

Studies have shown increased mortality in patients suffering stroke in cardiac surgery. According to Mahmoudi et al.^[10]^, the mortality of patients undergoing CABG and those who develop stroke within 24 hours is 25%. Dacey et al.^[15]^ evaluated patients undergoing CABG and observed that the survival rate in patients over 10 years was 26.9% in patients who suffered perioperative stroke and 61.9% in patients without stroke.

The literature has several articles addressing stroke and coronary surgery, but few including other types of surgery, especially valve surgery, as well as relating stroke in non-coronary and carotid stenosis surgery.

This study aims to show the main risk factors for ischemic CVA during and after surgery in patients undergoing coronary and non-coronary heart surgery in the Santa Casa de Misericórdia de Ponta Grossa-PR.

## METHODS

### Type of study

After approval by the Research Ethics Committee in humans at the Universidade Estadual de Ponta Grossa - Paraná, Brazil, Opinion No. 172,980, an analytical observational prospective case-control was developed in order to assess the influence of risk factors for the development of perioperative ischemic CVA in coronary and non-coronary heart surgery.

### Inclusion and exclusion criteria

Inclusion: from January 2010 to December 2012 all patients undergoing coronary and non-coronary heart surgery were prospectively evaluated in the Heart Surgery Service of Santa Casa de Ponta Grossa.

Exclusion: patients who did not undergo preoperative carotid Doppler or those with incomplete data were excluded.

It is routine in the Service performing carotid Doppler in all patients with coronary artery disease and in the other since they are 40 years or more.

### Prospective database

It was created a prospective database with the preoperative, intraoperative and postoperative information below, which allow compose the database of the EuroSCORE^[16]^, and other variables related to neurological complications, focus of this study. The researchers collected data directly from patients and examinations of electronic medical records evaluated daily during hospitalization of each patient.

### Preoperative data

Age, divided into two groups: under 70 years and 70 or more as other studies^[17,18]^; the characteristics of the EuroSCORE database were included as determining of that study^[16]^: gender; renal divided into four groups: (i) considered normal when creatinine clearance (CC) is greater than 85 ml/min, (ii) moderate dysfunction when CC higher than 50 ml/min and less than 85 ml/min (iii) severe dysfunction when CC less than 50 ml/min and finally (iv) patient under dialysis; poor mobility caused by neurological or skeletal muscle; poor mobility caused by neurological or muscle disorders; previous heart surgery; chronic lung disease, when the individual is under long-term treatment with steroids or bronchodilators for lung disease; active endocarditis; critical preoperative state including ventricular tachycardia or ventricular fibrillation or aborted sudden death, preoperative cardiac massage, mechanical ventilation before anesthesia, preoperative inotropic or intra-aortic balloon and acute renal failure (anuria or oliguria <10 ml/h); diabetes mellitus under insulin dependence or not; NYHA functional class; presence of class IV angina.

Left ventricular function divided into four classes: (i) good, when the ejection fraction (EF) was greater than 50%, (ii) moderate, when EF from 31% to 50%, (iii) poor, when EF from 21% to 30%, and finally (iv) very bad when EF less than 20%; acute myocardial infarction (AMI) in the last three months; pulmonary hypertension, classified as moderate when the systolic blood pressure in the pulmonary artery was between 31 mmHg and 55 mmHg and severe when above this last value; extracardiac arteriopathy: considered positive when the patient presents claudication, amputation for arterial disease and early or planned intervention in the abdominal aorta or arteries of the lower limbs; the presence of carotid lesion was studied separately from other peripheral vascular lesions and is considered as an isolated variable, as described below.

All patients with coronary artery disease and all patients were forty years old or more in any surgery routinely performed carotid Doppler at the same institution by a group of six different examiners, except where the urgency of the procedure did not allow enough time for the exam. The carotid examinations were classified from normal up to different degrees of obstruction, according to criteria of ECST (European Carotid Surgery Trial)^[19]^, which measures the residual lumen at the point of maximum degree of stenosis in the internal carotid artery and compared with what would be normal outer limit of the vessel in the same site of obstruction. For the statistical calculation of this sample were considered two groups of patients with or without major CS. Major carotid stenosis was defined as blocking 70% or more in the internal or common carotid on at least one side^[9,10]^. Patients with important CS had their mean arterial pressure (MAP) maintained above 70 mmHg perioperatively.

Left ventricular (LV) function was assessed by echocardiography in most patients, thus being the ejection fraction calculated by Teicholz, when there was no segmental dysfunction and Simpson in segmental dysfunction or significant change in ventricular geometry. In some patients with coronary artery disease was performed only ventriculography to assess LV function, especially in those with normal function and no segmental deficit.

### Trans-operative data

Degree of urgency of the surgical procedure divided into four groups: (i) elective, when the patient was admitted for routine operation, (ii) emergency, when the patient was not routinely elected for the operation, but needs intervention or surgery on the same admission for medical reasons, (iii) emergency, when the surgery was performed before the next working day (iv) rescue, when the patient required cardiopulmonary resuscitation on the road to surgery or before the induction of anesthesia; type of surgery performed: CABG, atrial septal defect correction, aortic surgery, left ventricular aneurysm, myxoma resection, or combined procedures and whether CPB was used or not, CPB time, aortic clamping time and use of vasoactive drugs.

### Postoperative data

Incidence of stroke and mortality. The stroke research was performed by daily review of electronic medical records by researchers and considered as ischemic stroke the presence of clinical changes longer than 24 hours compatible with ischemic injury associated with changes in cranial tomography (CT) as adopted by other authors, wherein the symptoms may have been present already in the recovery from anesthesia indicating an event intraoperatively or during the first postoperative hours, or later when the patient is recovering from anesthesia and subsequently presenting a neurological event after surgery^[10,20]^. The CT scan was performed immediately after the onset of symptoms, to rule out hemorrhagic event and repeated at 24 and 48 hours or more, when there was a change in the neurological status.

### Study groups and studied variables

Patients with diagnosis of ischemic stroke were allocated to group with IS (G_IS_) and the other patients in the control group (G_Control_). The criteria available in the database: gender, age, carotid stenosis, diabetes on insulin, chronic pulmonary disease, peripheral artery disease, renal function, unstable angina, left ventricular (LV) function, recent acute myocardial infarction, pulmonary arterial hypertension (PAH), use of cardiopulmonary bypass (CPB) were tested as risk factors for ischemic stroke. The mortality rate was also evaluated between groups.

### Statistical Analysis

Through statistical analysis, the variables to assess possible risk factors for ischemic stroke were tested. Continuous variables are presented as mean and standard deviation and nominal variables in absolute number and percentage, the distributions were tested for normality. To compare the means we used the Student' t test and to compare categorical variables the chi-square two-tailed test with Yates correction and logistic regression. For variables with values less than 5 we used for comparison the Fisher exact test. Were considered significant *P* values <0.05 and odds ratio ≥2.

### Description of the sample

The medical records of 626 patients were assessed, of which 73 were excluded for lack of preoperative carotid Doppler for being out of the examination protocol (less than 40 years in non-coronary surgery or urgent surgery with no time to perform Doppler) and 34 were excluded due to missing data. Resulting in a final sample of 519 patients after completion of the aforementioned exclusions.

## RESULTS

In the total sample was observed a prevalence of 4.2% (22) of perioperative ischemic stroke, being 17 in CABG, rate of 4.6% and 5 in non-coronary surgery, prevalence of 3.8%.

Through analysis of the sample population, it was found that the mean age was of 61.82 years, with 202 (38.92%) female and 317 (61.08%) males. Carotid stenosis of 70% or more occurred at 3.47% of the patients and 50% or more in 9.63%. For patients with perioperative ischemic stroke the mean age was 64 years, 9 (40.9%) patients were female and 13 (59.1%) were male, 10 (45.6%) had diabetes on insulin, 3 (13.63%) had CS greater than 70%; 9 (40.9%) had peripheral artery disease and 18 (81.8%) patients underwent surgery with CPB. Of the 22 cases of ischemic stroke observed: 3 (13.63%) occurred on the same side of the major carotid lesion, 12 (54.5%) occurred in the absence of any carotid lesion, 5 (22.7%) occurred in the presence of carotid lesion less than 70% and 2 (9%) patients had prior carotid endarterectomy. The average EuroSCORE of patients with ischemic stroke was 13.99 and for the control was 10.2. The other characteristics of the sample are presented in Table 1.

**Table 1:** Basic characteristics of the analyzed sample.

	Patients n=519 (%)
Gender	
Male	317 (61.08)
Female	202 (38.92)
IS	
Present	22 (4.24)
Absent	497 (95.76)
Age in years	
Mean	61.82±10.84
≥70	143 (27.55)
<70	376 (72.45)
Carotid stenosis	
≥70% in at least one side	18 (3.47)
No stenosis	501 (96.53)
≥50% in at least one side	50 (9.63)
No stenosis	469 (90.37)
Diabetes on insulin	
Yes	130 (25.05)
No	389 (74.95)
Chronic obstructive pulmonary disease	
Yes	149 (28.71)
No	370 (71.29)
Peripheral arterial disease	
Yes	113 (21.77)
No	406 (78.23)
Renal function	
Normal, creatinine clearance (CC)≥85	199 (38.34)
Moderate dysfunction (85<CC>50)	256 (49.33)
Severe dysfunction (CC<50)	57 (10.98)
Dialysis (CC<15)	7 (1.35)
Unstable angina	
Yes	264 (50.87)
No	255 (49.13)
Left ventricular function	
Normal ejection fraction (EF)≥ 0%	321 (61.85)
Moderate change (EF 30 to 50%)	133 (25.63)
Severe change (EF<30%)	65 (12.52)
Recent acute myocardial infarction	
Yes	177 (34.1)
No	342 (65.9)
Pulmonary arterial hypertension	
Yes (≥60 mmHg)	38 (7.32)
No (<60 mmHg)	481 (92.68)
Cardiopulmonary bypass	
Yes	395 (76.11)
No	124 (23.89)
CABG	
Yes	383 (73.8)
No	136 (26.2)
Mean EuroSCORE	
ICVA	13.99
Control	10.2
Outcome	
Discharge	437 (84.2)
Death	82 (15.8)
CABG	n=383 (100)
On-pump	260 (67.89)
Off-pump	123 (32.11)

CABG=coronary artey bypass surgery; IS=ischemich stroke

The prevalence of risk factors for the event was compared between groups and is shown in Table 2. Of the factors analyzed those statistically significant were as follows: carotid stenosis of 70% or more (*P*=0.03; OR 5.07; CI 95% 1.35 to 19.02), diabetes mellitus using insulin (*P*=0.04; OR 2.61; CI 95% 1.10 to 6.21) and peripheral artery disease (*P*=0.03; OR 2.61; CI 95% 1.08 to 6.28).

**Table 2:** Risk factors analyzed for the development of postoperative CVA.

	G_icva_	G_Control_	*P*
	n=22	n=497	
Gender			
Male	13	304	
Female	9	193	0.82
Age (years)*			
Mean	64.13±8.1	61.72±10.42	0.28
>or=70	6	121	
<70	16	376	0.75
Carotid stenosis*			
≥70% unilateral	3	15	
No stenosis	19	482	0.03
≥50%	5	45	
No stenosis	17	452	0.05
Diabetes on insulin			
Yes	10	120	
No	12	377	0.04
Chronic obstructive pulmonary disease			
Yes	6	143	
No	16	354	1.000
Peripheral arterial disease			
Yes	9	104	
No	13	393	0.03
Renal function			
Normal	6	193	
Slight	11	245	
Moderate	5	52	
Severe	0	7	0.26
Renal function			
Normal	6	193	
Changed	16	304	0.28
Unstable angina			
Yes	14	250	
No	8	247	0.27
Left ventricular function			
Normal	13	308	
Moderate change	7	126	
Severe change	2	63	0.74
Recent acute myocardial infarction			
Yes	8	169	
No	14	328	0.82
Pulmonary hypertension*			
Yes	1	37	
No	21	460	0.92
Cardiopulmonary bypass*			
Yes	18	377	
No	4	120	0.69
CABG			
Yes	17	366	
No	5	131	0.80
Outcome			
Discharge	16	421	
Death	6	76	0.13
CABG*	n=17	n=366	
On-pump	13	247	
Off-pump	4	119	0.61

CABG=coronary artey bypass surgery; CVA=cerebrovascular accident.

* Variables analyzed by the Fisher ’s exact test.

## DISCUSSION

Most studies address the incidence of stroke in patients undergoing coronary surgery, one of the few reports that addresses different surgical procedures with large case series was developed by Bucerius et al.^[21]^ who analyzed 16,184 patients and found 4.6% of ischemic events, but included in the sample not only stroke patients, but also those with transient ischemic attack; in the present study the incidence of ischemic events was similar to the above study (4.2%), however the samples are different in two aspects, first, here were considered only cases of ischemic stroke confirmed by clinical changes accompanied by change on cranial CT - as criterion adopted by other studies^[10,20]^, and second: we excluded patients without coronary artery disease under 40 years and therefore lower incidence of stroke. It was not found in the widely researched literature a study with identical design to compare the incidence of stroke, and the article by Bucerius et al.^[21]^ was the most closely.

The exclusion of patients undergoing coronary surgery and not less than 40 years was performed because these patients do not undergo carotid Doppler, avoiding their inclusion in the statistics, on the other hand, the patients with valvular or congenital diseases with 40 years or more were included since there was lack of publications on the occurrence of stroke in this population, especially correlated with carotid disease. In Brazilian Services living with degenerative valve disease and with a large number of rheumatic diseases, it is worth a look at the causes of neurological ischemic events in this population, given the large number of valve surgeries herein. In this series the prevalence of ischemic stroke in non-coronary surgery was not statistically different in the incidence of ischemic stroke in CABG, showing the relevance of studying also the risk factors for stroke in this population.

Although age is mentioned as a risk factor for perioperative stroke in some studies, in this sample, age greater than 70 years was not correlated statistically with higher incidence of stroke. The average age of patients with stroke was three years older than the control average, but without significant differences, showing that other factors such as degree of patient's atherosclerosis demonstrated by the presence of peripheral artery disease, severe carotid CS and DM on insulin seem to be more relevant than age alone as risk factors for stroke in this sample. Studies with larger samples as Bilfinger et al.^[17]^, who evaluated 2071 patients and Gardner et al.^[18]^, who evaluated 3279 patients, observed a correlation between increased age and increased risk of stroke. An increase of this series, which continues to be expanded, may in future studies to correlate again age and stroke with greater statistical power.

According Inzitari et al.^[9]^, asymptomatic carotid stenosis 60-99% are at risk of stroke of 16.2% in five years. Hirotani et al.^[20]^, correlating ischemic stroke with cardiac surgery found rates of 2.7% in patients with carotid stenosis <50%, and 16.7% in patients with carotid stenosis ≥50%. In this series, there was a 10% rate of ischemic stroke in patients with carotid stenosis ≥ 50%, and in carotid stenosis ≥ 70% this index reaches about 16.6%. But it is still debatable the influence of carotid stenosis in the pathophysiology of perioperative ischemic stroke in cardiac surgery, given that patients who have carotid atherosclerosis possibly have severe atherosclerosis of the ascending aorta, which is one of the biggest risk factors for ischemic stroke^[2,22]^. In this sample, the probability that a patient who had suffered ischemic stroke presenting severe stenosis was 83%, and even though the carotid stenosis greater than 70% has been found as an important risk factor for ischemic stroke, of the 22 events, only 3 (13.63%) showed a correlation between the side of the carotid lesion and the side affected by ischemic injury, which is compatible with data published by Roffi et al.^[2]^ affirming that the prevalence of ipsilateral stroke in patients with asymptomatic lesion and from 50 to 99% was only 2%. This evidence strongly suggests that carotid lesions are more a risk marker than actually the etiology of stroke, this creates great doubt about the benefits of prophylactic treatment of carotid lesions in patients without neurological symptoms. Multicenter randomized studies comparing prophylactic strategies of endarterectomy, carotid angioplasty and no prophylactic treatment, taking into account the degree of stenosis and the presence of unilateral or bilateral disease, could clarify which is the best approach in this group of patients.

The CPB is recognized as a potential risk factor for stroke in cardiac surgery especially by increasing the manipulation of the aorta^[23]^, the present study found a risk 1.4 times higher in patients undergoing CPB, but without statistical difference. A larger sample could in the future demonstrate that off-pump surgery is capable of reducing the incidence of stroke, as shown in meta-analyzes^[13,14]^.

Besides plaques in the aorta and extracranial carotid stenosis, other factors interfere with cerebral perfusion, for example intracranial atherosclerosis, arterial pressure and therefore maintain adequate oxygenation is necessary to avoid extended shock or severe hypoxemia that may lead to lesions brain, in the Service it is practice maintaining mean arterial pressure above 70 mmHg in patients with higher risk of stroke, especially in patients with significant carotid disease in patients with surgery with or without cardiopulmonary bypass.

It is known that diabetes mellitus, despite being a potentially treatable factor, may be the second largest risk factor for ischemic stroke and its incidence has been increasing in the last decade^[24]^. In this study, diabetes using insulin was a risk factor for ischemic stroke corroborating findings of other authors^[2,15,21]^. Diabetes, despite being an important marker of more severe atherosclerosis of large vessels, affects the microcirculation and interferes significantly with endothelial reactivity, damaging tissue perfusion as a whole and predisposing still other possible complications to lead to low output and consequent cerebral ischemia as greater degree of myocardial ischemia, greater inflammatory response and more prone to infection.

It was observed in the present series, that the presence of peripheral artery disease was more prevalent in the group with ischemic stroke that comes against the finding of other studies^[7,20]^. It is clear from the evidence presented herein that the same risk factors for stroke are the markers of most severe systemic atherosclerosis as the presence of diabetes using insulin, severe stenosis of the carotid and peripheral artery disease, and should always serve as a warning to the surgeon in the increased care with this group of patients at high risk for stroke. A patient with the above three features together have 33.8 times more chance of a perioperative ischemic stroke when compared to a patient without these characteristics.

Unlike studies by Mahmoudi et al.^[10]^ and Dacey et al.^[15]^, in which the presence of stroke increased mortality, in this study, there was no statistical difference in the mortality rate in patients with and without ischemic stroke, which can be related to the sample size.

## CONCLUSION

In this sample the significant risk factors for perioperative ischemic stroke in cardiac surgery were: carotid stenosis equal to or greater than 70%, presence of diabetes on insulin and presence of peripheral artery disease.

**Table:** 

**Authors’ roles & responsibilities**	
MACC	Analysis and/or interpretation of data; statistical analysis; final approval of the manuscript; study design; implemen-tation of projects and/or experiments; writing of the manu-script or critical review of its content
MFG	Analysis and/or interpretation of data; writing of the manu-script or critical review of its content
RZG	Final approval of the manuscript; study design; writing of the manuscript or critical review of its content
MDS	Analysis and/or interpretation of data; statistical analysis
